# A Novel Application of Phosphorene as a Flame Retardant

**DOI:** 10.3390/polym10030227

**Published:** 2018-02-26

**Authors:** Xinlin Ren, Yi Mei, Peichao Lian, Delong Xie, Yunyan Yang, Yongzhao Wang, Zirui Wang

**Affiliations:** 1 Faculty of Chemical Engineering, Kunming University of Science and Technology, Kunming 650500, Yunnan, China; ren8877@126.com (X.R.); cedlxie@kmust.edu.cn (D.X.); 15216105302@163.com (Y.Y.); wyz201610810119@126.com (Y.W.); kgwzr2014@163.com (Z.W.); 2 The Higher Educational Key Laboratory for Phosphorus Chemical Engineering of Yunnan Province, Kunming University of Science and Technology, Kunming 650500, Yunnan, China

**Keywords:** phosphorene, black phosphorus, flame retardant, polymer, waterborne polyurethane

## Abstract

Black phosphorene-waterborne polyurethane (BPWPU) composite polymer with 0.2 wt % of black phosphorene was synthesized. Scanning electron microscopy (SEM) was used to observe the morphology of phosphorene in polyurethane matrix, which indicated that the phosphorene distributes uniformly in the PU matrix. The flammability measurements were carried out to investigate the flame-resistant performances of phosphorene, which indicated that phosphorene could effectively restrict the degradation of the PU membrane. Compared by the pure WPU, the limiting oxygen index (LOI) of BPWPU increased by 2.6%, the heat flow determined by thermal analysis significantly decreased by 34.7% moreover, the peak heat release rate (PHRR) decreased by 10.3%.

## 1. Introduction

Monolayer or several-layer black phosphorus (BP), also named black phosphorene or phosphorene, is a type of two-dimensional (2D) nanomaterial with distinct physical/chemical properties due to the dimensionality effect. Phosphorene can be exfoliated by mechanical exfoliation [1,2], liquid phase exfoliation [3,4,5], or some other methods [6,7] from bulk BP due to the fact that the BP has a layered structure, in which weak interaction enables stacking of layers and the strong covalent bond holds atoms together in plane [8]. Phosphorene has a direct band gap tunability from 0.3 to 2.0 eV, a higher carrier mobility (1000 cm^2^·V^−1^·s^−1^) at room temperature, a high on-off ratio (10^5^), strongly anisotropic feature, as well as good current saturation in field-effect-devices [9,10,11,12,13,14]. These excellent properties make phosphorene a great candidate for electronic, photonic, medical, and thermoelectric devices. 

Red phosphorus and some phosphorus-containing compounds have been used as flame retardants in the early past, because they can be formed into phosphoric acid by high temperature thermal decomposition [15], which promotes the polymers to form into heat-resistant carbonaceous protective layer, consequently interferes with the transport of oxygen to the burning zone [16]. Phosphorus may also react with H or OH radicals, to reduce the energy of the flame in the gas phase [17]. However, most of the phosphorus-containing flame retardants are demanded to add a large proportion in order to improve the flame retardant performance, and they have a low compatibility between flame retardant and matrix materials [18]. It is interesting to find that phosphorene is a type of 2D nanomaterial, which has the same feature as the other nano flame retardants, such as graphene [19,20,21,22,23,24] and carbon nanotubes [25,26,27], leading to an high flame resistant efficiency with a low additive amount. They can also distribute uniformly into matrix materials and qualify a good compatibility with polymers due to the small size. Thus, phosphorene is expected to be an efficient flame retardant. In this paper, we report a novel application of black phosphorene as a flame retardant for polymers. We choose waterborne polyurethanes (WPU) as the matrix polymer since WPU have been widely used in the films, adhesives, paints, varnishes, and coatings, but they are inflammable, leading to an non-negligible disadvantages that threatens people’s safety [28,29,30].

## 2. Materials and Methods 

### 2.1. Materials

In the present study, the BP was prepared by mineralization transformation method (as shown in Section 2.2). The red phosphorus (RP), iodine (I), tin (Sn), toluene, and acetone were analytically pure. The water was deionized water. The WPU latex (Anhui Huatai New Material Co. Ltd., Hefei, China) was purchased with an effective solid content of approximately 30 wt %.

### 2.2. Synthesis of BP Crystals

Firstly, SnI_4_ was synthesized by mixing iodine and tin into a toluene solution after heat reflux condensation. RP (1000 mg), Sn (240 mg), and SnI_4_ (60 mg) were sealed in a quartz tube under argon atmosphere. The sealed tube was placed horizontally in the reaction zone of a tube furnace. Then the furnace was firstly heated to 650 °C within 1 h and then kept at 650 °C for 24 h. The furnace was then cooled to 500 °C at a rate of 40 °C·h^−1^ and held at 500 °C for 30 min. After being cooled to room temperature, the large BP crystals were formed. Finally, the BP crystals were washed with toluene to remove the residual mineralizer, followed by water and acetone washing.

### 2.3. Preparation of Phosphorene

The BP were ground for 2 h into powders, and then the powders were added into the deionized water with an initial solid-to-liquid ratio of 1 mg:1 mL. A type of suspension liquid was formed by sonication for 24 h in the ultrasonic bath (50 Hz, 200 W), which was centrifuged at 3000 rpm for 30 min by a centrifugal machine (TGL-16C, Shanghai Anting Scientific Instrument Factory, Shanghai, China). Finally, the supernatant liquid containing phosphorene was collected and condensed in a glass bottle. An argon atmosphere should be used to prevent the oxidation of phosphorene in the experimental process. The content of solid phosphorene in the obtained suspension liquid was 40 mg/L.

### 2.4. Preparation of Black Phosphorene-WPU (BPWPU) Composite Materials 

**Figure 1:**
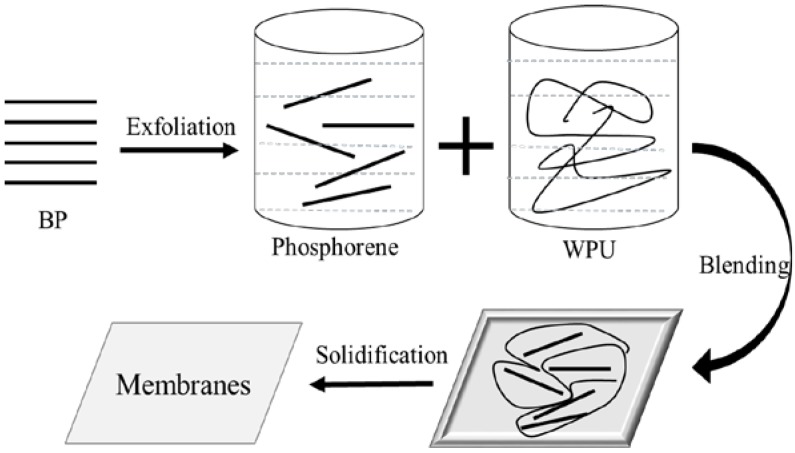
Preparation flow diagram of the composite membrane.

Sixty grams of WPU latex and 1 L of phosphorene dispersion were added into a beaker. After stirring for a few minutes, the beaker was sealed with an argon atmosphere filling. Then the mixed suspension liquid was put into the ultrasonic device for 2 h. The temperature was maintained not to surpass 30 °C by using an ice bath. Then the obtained suspension liquid was poured into a square plate with the size of 240 × 300 mm and dried into a vacuum drying oven at room temperature. A few days later, the film of the BPWPU composite material with a thickness of approximately of 0.5 mm was formed. The preparation flow diagram to synthesize the BPWPU composite membrane is shown in Figure 1.

### 2.5. Analytical Procedure

#### 2.5.1. Structure Characterizations

A fraction of bulk BP was ground into powders and then transferred on a glass slide for X-ray diffraction (XRD, PANalytical Empyrean, Almelo, The Netherlands) measurement. It was carried out at room temperature on a PAN analytical X-ray diffractometer equipped with a monochromatic CuK α1 radiation and 40 kV of voltage. 

After the phosphorene was dried under inert atmosphere, it was observed by transmission electron microscopy (TEM, Philips, Amsterdam, The Netherlands) with a Philips CM100 apparatus using an acceleration voltage of 100 kV. 

Scanning electron microscopy (SEM, Bruker Nano, Bruker, Karlsruhe, Germany) was taken on the BP powders to detect the layered structure of BP. It was also taken on the fracture surfaces of polymers by liquid nitrogen. The fracture surfaces of the specimens were coated with a conductive layer of gold powders prior to testing. In addition, in order to present the distribution of phosphorene in the polymers, mapping was carried out on another two specimens without the coating of a gold layer. 

The Fourier transform infrared spectroscopy with a temperature-controlled attenuated total reflection device (FTIR-ATR, Bruker-Vertex 70, Bruker, Karlsruhe, Germany) and X-ray photoelectron spectroscopy (XPS, PHI5000 Versaprobe-II, ULVAC-PHI, Tokyo, Japan) at 15 kV and 50 W were conducted on the samples to characterize the structural and chemical bonding of phosphorene in polymers.

#### 2.5.2. Thermal Properties Measurement

Thermogravimetric analysis (TGA) and differential scanning calorimetry (DSC) were performed using a thermal analyzer (NETZSCH STA449F3, NETZSCH, Selb, Germany) at a heating rate of 20 °C/min and gas flow rate of 100 mL/min from room temperature to 800 °C in air atmosphere.

#### 2.5.3. Flammability Property Measurement

The limiting oxygen index (LOI) values were measured according to the standard oxygen index test of ISO 4589-2:1996 by using the device from Motis Combustion Technology Co. Ltd. (COI, Kunshan, China). 

The polymers were also performed by directly setting fire in ambient surroundings to analyze the flame resistant effect of phosphorene.

Microcombustion calorimetry (MCC) was performed on a Govmark MCC-2 microscale combustion calorimeter (Govmark, Farmingdale, NY, USA) according to ASTM D 7309-13 protocol. Five miligrams of samples were heated from 80 °C to 750 °C at a heating rate of 0.99 °C/s after being drying under 75 °C for 8 h. 

## 3. Results and Discussion

### Preparation and Characterization of BP and Phosphorene

**Figure 2:**
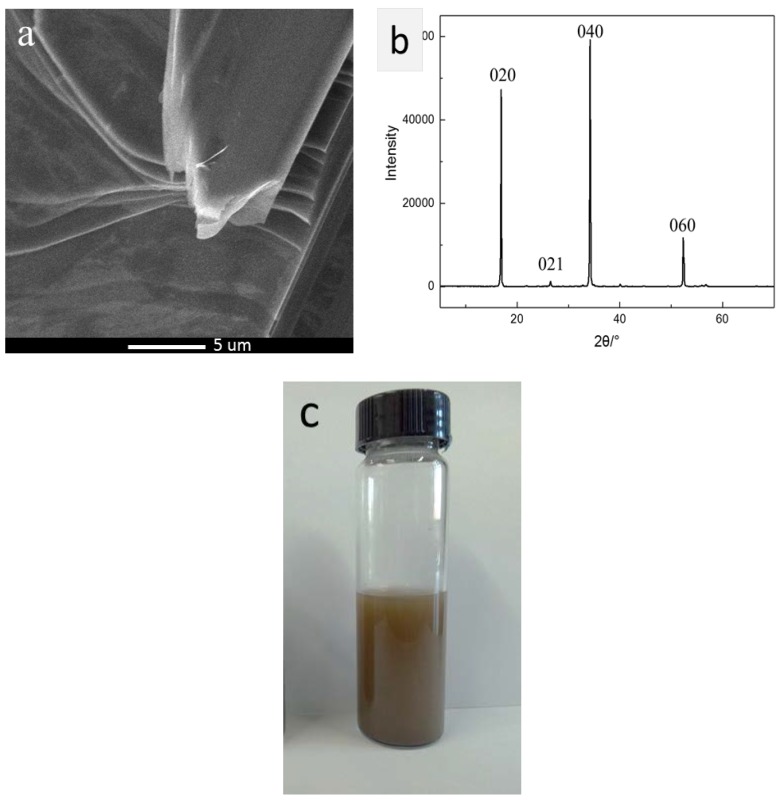

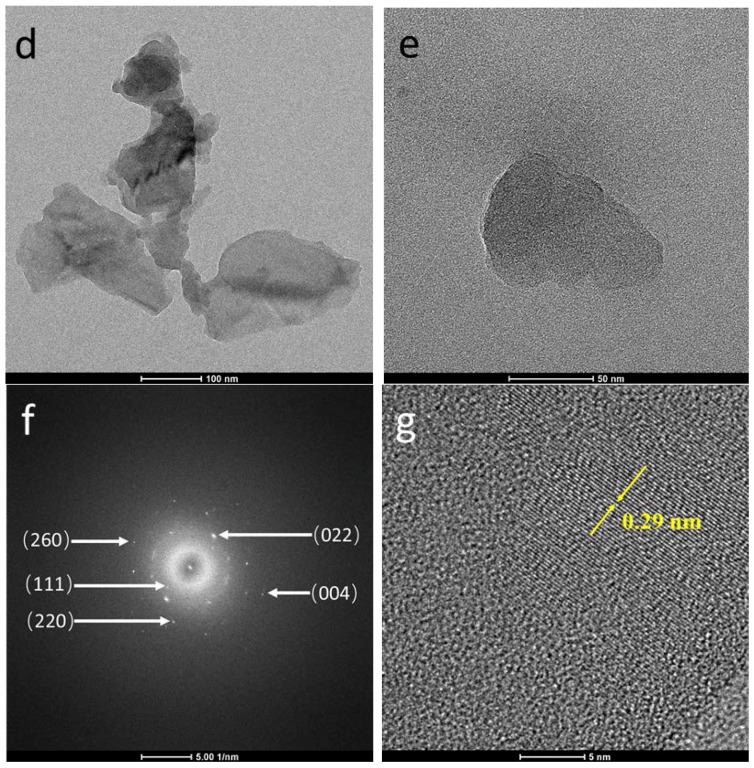
(**a**) SEM image of bulk BP; (**b**) XRD spectra of BP; (**c**) images of phosphorene dispersion in deionized water; (**d**,**e**) typical TEM images of phosphorene; (**f**) SAED pattern showing the orthorhombic crystal structure of the sample; and (**g**) high-resolution TEM image of monolayer phosphorene.

BP crystals were prepared from pure red phosphorus by mineralization transformation method with Sn and SnI_4_ as mineralization additives. The SEM image of BP (Figure 2a) showed that the BP has a layered structure. For further testifying the structure, X-ray diffraction (XRD) was carried out on the BP powders, as shown in Figure 2b, which indicated that the BP had a good crystallinity without impurities. Then the bulk BP crystals were ground to powders before being added into the deionized water to prepare phosphorene dispersion (see Figure 2c) by sonication and centrifugation. The structure of the obtained phosphorene was characterized using a transmission electron microscopy (TEM), and the results were shown as Figure 2d–g. Figure 2d,e showed the typical images of phosphorene, which indicated that the thin sheets of phosphorene had a size of 50–200 nm. Figure 2f,g showed the selected area electron diffraction (SAED) pattern and high resolution TEM image of the same mono-layer phosphorene, respectively. The SAED pattern recorded on this sample depicted the good crystalline of the phosphorene, and the high-resolution TEM images of phosphorene certified its layered or 2D structure with the interlayer spacing approximately 2.9 nm. These results were in good agreement with those in the early literatures [4,31,32]. Afterwards, the obtained phosphorene dispersion was added into WPU latex dispersion and dried in a vacuum drying oven to prevent it from contacting with air. At last, the thin BPWPU membrane with 0.2 wt % of phosphorene was synthesized. At the same time, the pure WPU membrane was prepared for comparison. The prepared membranes had a thickness of approximately 0.6 mm and showed transparent, smooth and self-sustained properties. Due to the addition of phosphorene, the BPWPU showed a little yellowish-brown color but still remained transparent.

**Figure 3:**
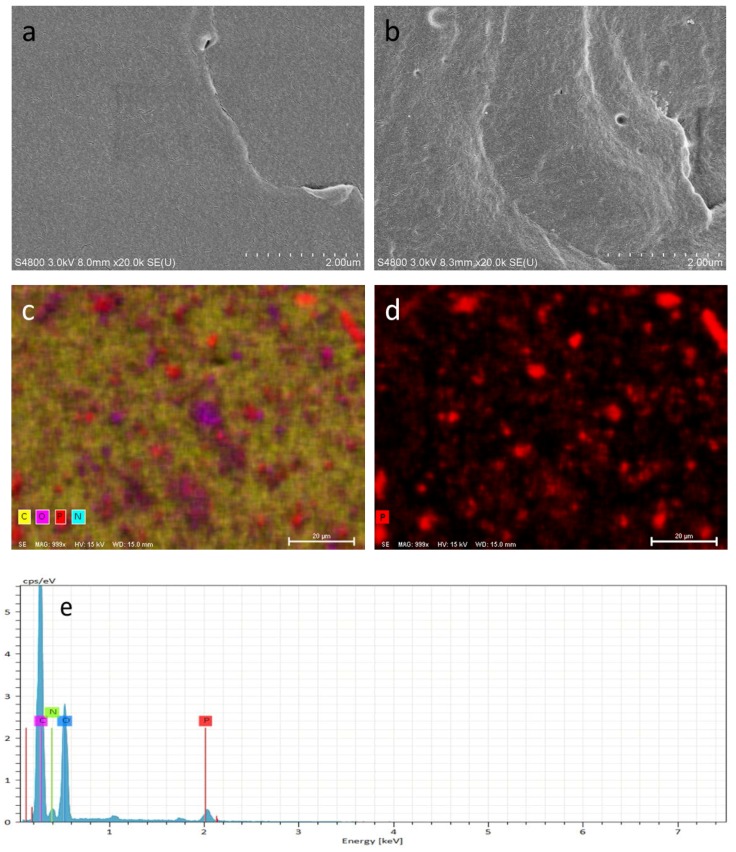
(**a**) SEM image of pure WPU; (**b**) SEM image of BPWPU; (**c**) mapping test of BPWPU; (**d**) mapping of phosphorene in BPWPU; and (**e**) EDS image of BPWPU.

The SEM images of the fracture surfaces could provide the information concerning the interfacial interactions between phosphorene and the matrix. The brittle fractured surfaces of pure WPU (Figure 3a) and BPWPU (Figure 3b) by liquid nitrogen were observed using SEM. The image of pure WPU presents homogeneous and smooth surface due to the typical fracture behavior of homogeneous material. In comparison of the blank WPU, the fractured surface of BPWPU composites is much rougher due to the addition of phosphorene. The image of BPWPU shows a lot of white pots, silks, or floccules, which were intertwining together uniformly. Mapping was conducted on the surface of BPWPU membrane (see Figure 3c,d), which showed that the four elements (C, N, O, P) interlaced with each other and a small amount of phosphorene (see as the red sheet areas) distributed uniformly in the specific zone. The result of energy dispersive spectrometer (EDS) presented a small peak of P (see Figure 3e), which testified the successful addition of phosphorene. 

**Figure 4:**
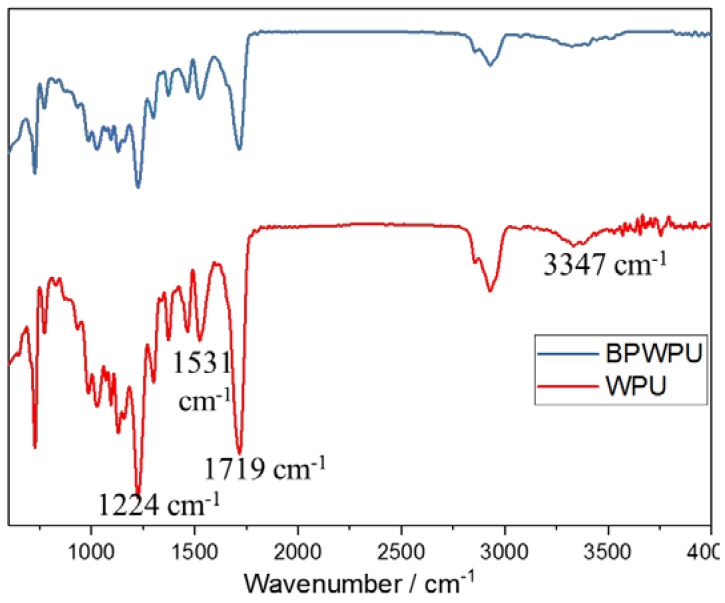
FTIR-ATR spectra of WPU and BPWPU membranes.

To investigate the interaction of phosphorene and PU, a Fourier transform infrared spectroscopy on a temperature-controlled attenuated total reflection device (FTIR-ATR) was utilized on the membranes. The results are displayed in Figure 4. It is found that these peaks revealed a typical structure of WPU, where the peaks at 1224 cm^−1^ is attributed to the stretching vibration of C–O–C in urethane bonding (–NHCOO–) [33] and 3347 cm^−1^ refers to urethane N-H stretching vibration with the corresponding deformation vibration peak appearing at 1531 cm^−1^ [34]. In addition, the peak of 1719 cm^−1^ [35] is the characteristic peak of –COOH, which is the hydrophilic group of WPU mentioned above. However, the characteristic peaks of phosphorus-based groups (i.e., stretching vibration of P–O–C at 1171 cm^−1^ and P=O at 1325 cm^−1^ [18]) have not been observed, indicating that the phosphorene have not reacted with the elements or functional groups in WPU to form into covalent bonding. Thus, phosphorene is an additive type of flame retardant agent, which combines with WPU through intertwining and wrapping.

**Figure 5:**
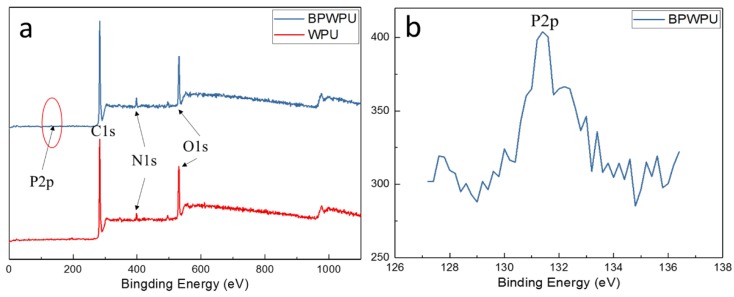
(**a**) XPS spectra of WPU and BPWPU membranes; (**b**) the enlarged image for the peak of P2p of BPWPU.

The structures of pure WPU and BPWPU were also investigated by X-ray photoelectron spectroscopy (XPS), as shown in Figure 5a, which offered more information about surface composition and chemical state of phosphorene in WPU. The XPS survey spectrum of BPWPU shows that the sample includes P, O, C, and N elements, also implying that the synthesized membrane contains phosphorene. It can be observed that C, N, and O peaks are the typical peaks, which are in good agreement with the early literatures [36,37,38]. The enlarged image (see Figure 5b) at the peak of P2pof BPWPU exhibits an increased intensity due to the addition of phosphorene. The appearance of P2p peak of BPWPU is originating from the additive phosphorene on the surface of the composite material. 

**Figure 6:**
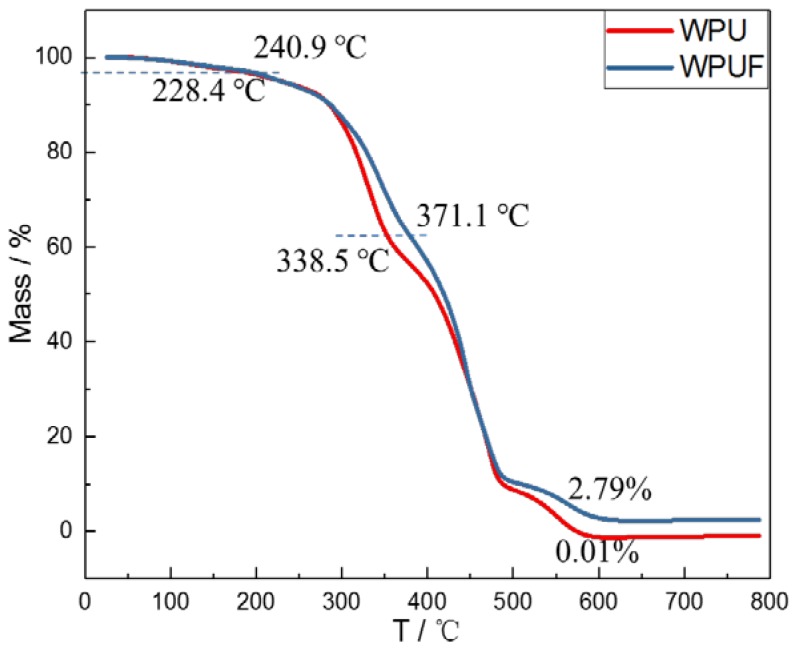
TGA curves of pure WPU and BPWPU membranes in the air atmosphere.

The resulted thermogravimetric analysis (TGA) curves of WPU and BPWPUF in the air atmosphere are shown in Figure 6. The pyrolysis curves of WPU and BPWPUF can be divided into three stages without an obvious boundary. The first mass loss stage take place no more than 240.9 °C, which is mainly caused by the volatilization of water adhere on the surface of materials. The major weight loss takes place in the second stage between 240.9 °C and 480 °C because the amine, isocyanate, and olefins generated from the breakage of urethane bonds. The third weight loss stage is assigned to the decomposition of residual polyols. As we can see, the initial decomposition temperature (5 wt % weight losses) of BPWPU (228.4 °C) takes place at lower temperatures than that of pure WPU (240.9 °C). The reason is that the phosphorene forms into phosphorous acid in combustion, and then the phosphorous acid promotes the thermal decomposition of polyurethane [18]. The weight loss rate of BPWPU becomes slower than that of WPU covering almost all the second stage, and the biggest gap, more than 30 °C, occurred in the middle of the second stage. After the third weigh loss stage, the materials are broken down into gases under a high temperature and air atmosphere, resulting in only a small part of residue chars. In addition, the residue chars of BPWPU (2.79%) is higher than that of the pure WPU, indicating that the phosphorene has transformed into polyphosphoric acid and promoted the formation of char layers, which can further retard the diffusion of flammable gases in the condensed phase. 

**Figure 7:**
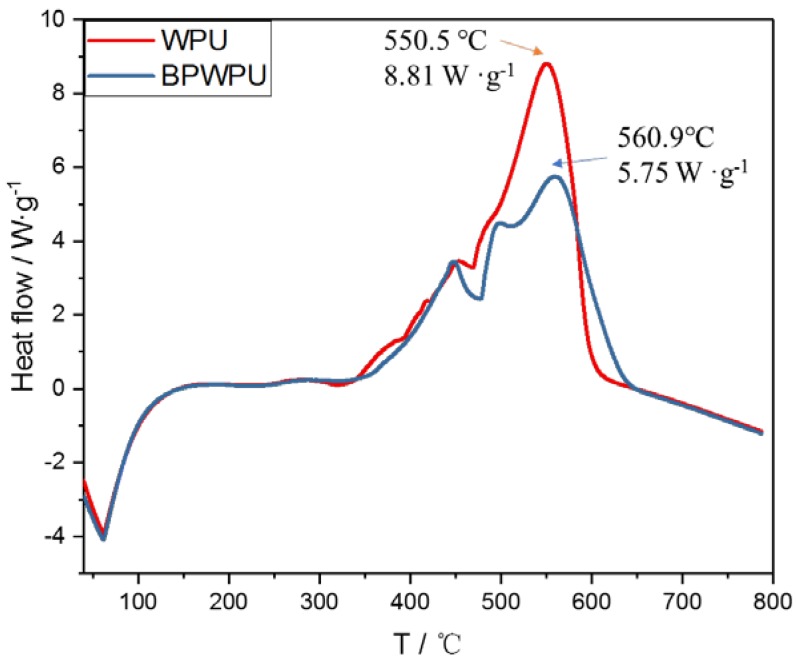
Heat flow curves of pure WPU and BPWPU membranes in the air atmosphere by using a DSC measurement.

Figure 7 shows the heat flow curves of pure WPU and BPWPU determined by DSC in the air atmosphere. The HRR curve of pure WPU has three different peaks. The middle one is not very significant and the third one is a very sharp peak, which corresponding to the typical combustion of WPU [39]. The peak heat flow of pure WPU appears at 26.4 min with a value of 8.81 W·g^−1^. The heat flow curve of BPWPU also appears three peaks. The first heat flow peak has little difference with the pure WPU. The second one is a new peak and it is obviously lower than the value of pure WPU. The third peak is the highest peak of BPWPU, which indicates that the peak heat flow of BPWPU is 34.7% lower than that of the pure WPU. Additionally, the heat flow curve of pure WPU decreases quickly after the last peak, while the heat flow curve of BPWPU decreases slower and keeps a longer time before the BPWPU are decomposed completely. These results indicate that phosphorene has a good heat inhibiting effect, which can effectively improve the flame-resistant performance of polymers.

The limiting oxygen index (LOI) test was performed to determine the flammability of the two films. As depicted in Table 1, the pure WPU is an inflammable polymeric material with a LOI of 21.6%, and the LOI of BPWPU (24.2%) has been obviously increased by 2.6% by the introduction of phosphorene. There are two key reasons for the high LOI value of BPWPU. On the one hand, the addition of phosphorene has significantly improved the residual char according to the TGA results, which not only have the heat insulation function, but can also prevent the flame propagation, restrain the drip, and reduce the smoke generation. That is to say, it enhances the self-extinguishment of WPUs. On the other hand, new noncombustible gas ammonia is produced by introducing of phosphorene, and the oxygen concentration surrounding WPU decreases due to the dilution of ammonia, which may have the flame retardant effect. Additionally, this non-combustible gas also has the effect of radiation and heat dissipation. These results are consistent with other literature [33,40,41]. In all, LOI testing result show a significant improvement in fire resistance in the presence of phosphorene in WPU polymers.

**Figure 8:**
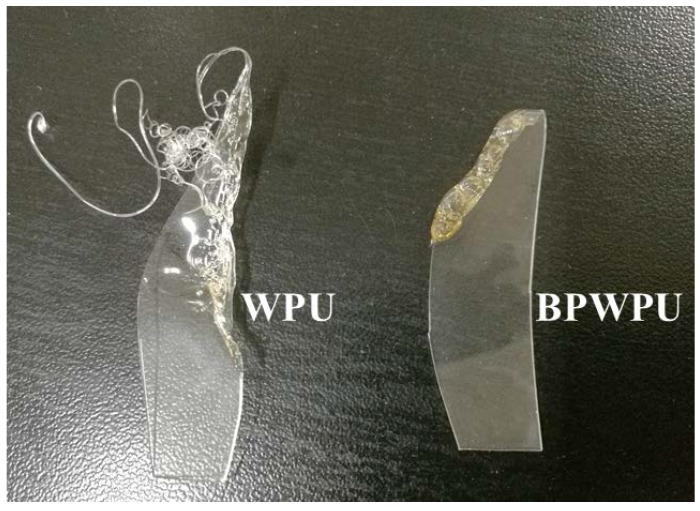
Burning surface images of the pure WPU (**left**) and BPWPU (**right**) strips in ambient surroundings.

Combustion tests were performed in ambient environment to examine the truly burning situation of pure WPU and BPWPU. It is found that the pure WPU is easy to spunk, and then it burns quickly with a burning time approximately of 15 s as the WPU strip burns more than 5 cm of the length, as shown in Table 1. On the contrary, the pure BPWPU is relatively difficult to spunk. In addition, the BPWPU strip self-extinguishes two times when it is burning. The total burning time of the BPWPU strip was approximately 25 s. Figure 8 shows the burning surface images of the pure WPU (left) and BPWPU (right) strips. The two burning surfaces were significantly different as the pure WPU produced many threadlike residues, while the burning surface of BPWPU was flat and orderly. That is to say, the pure WPU produces large amount of molten drops when it is burning, which will not occur on BPWPU due to the presence of phosphorene. As for pure WPU, there is no obvious formed char layer because the WPU almost decomposes completely. However, it can be observed that the surface morphology of the char layer for BPWPU presents a relatively compact structure on the surface due to much char formation during combustion, therefore, the flame, heat, and flammable volatiles could be prevent by the char layer into the inner zone. The results indicate that the incorporation of phosphorene promotes the formation of a char layer with higher thermal stability, which can restrict the transfer of heat and flammable volatiles and leading to excellent flame retardancy. 

**Figure 9:**
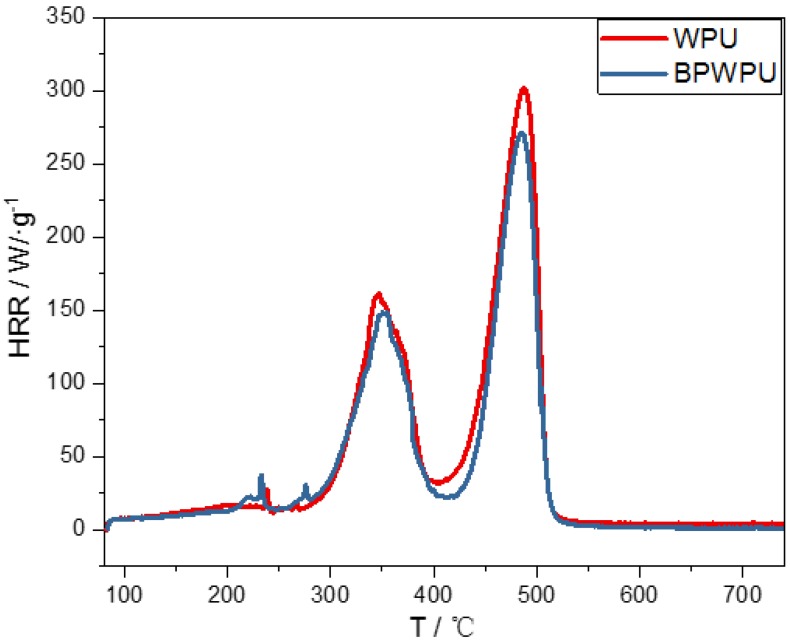
The HRR curves from cone calorimeter tests.

The heat release rate (HRR) is recognized to be the most important parameter to evaluate the flame retardant. Figure 9 shows the heat release rate (HRR) curves of pure WPU and BPWPU composite material determined by MCC. The HRR curves shows two sharp peaks due to the typical two combustion stages of the WPU materials. Pure WPU burns rapidly after ignition and HRR shows a peak heat release rate (PHRR) of about 302 W·g^−1^ at 487.5 °C. Comparably, the PHRR of BPWPU is 271 W·g^−1^, which decreased by 10.3%. The residue char of WPU and BPWPU were 1.7% and 2.3%, respectively. The significantly reduction in the PHRR can be attributed to the phosphorene being active via flame inhibition and char formation. These results are consistent with the results of TGA and DSC.

## 4. Conclusions

We have demonstrated a novel application of black phosphorene as a flame retardant by adding a small amount of it into WPU polymer. Phosphorene can effectively improve the thermostability, flame-resistance, and LOI of WPU; moreover, it can significantly decrease the HRR and restrict the degradation of the WPU. The results indicate that the black phosphorene is an excellent flame retardant, which not only can distribute uniformly into the WPU polymer, but also displays outstanding flame-resistant performances. This work develops a novel application of phosphorene as flame retardant for improving the performance of polymers, which would pave the way for the applications of nano BP in flame retardant field.

## Figures and Tables

**Table 1:** Comparison of the LOI and burning time.

Name	LOI/%	Burning Time/s
Pure WPU	21.6	15
BPWPU	24.2	25
